# The Role of the Neutrophil to Lymphocyte Ratio for Survival Outcomes in Patients with Metastatic Castration-Resistant Prostate Cancer Treated with Abiraterone

**DOI:** 10.3390/ijms18020380

**Published:** 2017-02-11

**Authors:** Martin Boegemann, Katrin Schlack, Stefan Thomes, Julie Steinestel, Kambiz Rahbar, Axel Semjonow, Andres Jan Schrader, Martin Aringer, Laura-Maria Krabbe

**Affiliations:** 1 Department of Urology, Prostate Center, University Hospital Muenster, Albert-Schweitzer-Campus 1, GB A1, D-48149 Muenster, Germany; katrin.schlack@ukmuenster.de (K.S.); s_thom09@uni-muenster.de (S.T.); Julie.steinestel@ukmuenster.de (J.S.); axel.semjonow@ukmuenster.de (A.S.); andresjan.schrader@ukmuenster.de (A.J.S.); laura-maria.krabbe@ukmuenster.de (L.-M.K.); 2 Department of Nuclear Medicine, Muenster University Medical Center, Albert-Schweitzer-Campus 1, GB A1, D-48149 Muenster, Germany; kambiz.rahbar@ukmuenster.de; 3 Department of Rheumatology, Dresden University Medical Center, Fetscherstraße 74, D-01307 Dresden, Germany; martin.aringer@uniklinikum-dresden.de; 4 Department of Urology, University of Texas Southwestern Medical Center, Dallas, TX 75390-9110, USA

**Keywords:** neutrophil-to-lymphocyte ratio, castration-resistant prostate cancer, prognostic biomarker, treatment response, abiraterone

## Abstract

The purpose of this study was to examine the prognostic capability of baseline neutrophil-to-lymphocyte-ratio (NLR) and NLR-change under Abiraterone in metastatic castration-resistant prostate cancer patients. The impact of baseline NLR and change after eight weeks of treatment on progression-free survival (PFS) and overall survival (OS) was analyzed using Kaplan-Meier-estimates and Cox-regression. 79 men with baseline NLR <5 and 17 with NLR >5 were analyzed. In baseline analysis of PFS NLR >5 was associated with non-significantly shorter median PFS (five versus 10 months) (HR: 1.6 (95%CI:0.9–2.8); *p* = 0.11). After multivariate adjustment (MVA), ECOG > 0–1, baseline LDH>upper limit of normal (UNL) and presence of visceral metastases were independent prognosticators. For OS, NLR >5 was associated with shorter survival (seven versus 19 months) (HR: 2.3 (95%CI:1.3–4.0); *p* < 0.01). In MVA, ECOG > 0–1 and baseline LDH > UNL remained independent prognosticators. After 8 weeks of Abiraterone NLR-change to <5 prognosticated worse PFS (five versus 12 months) (HR: 4.1 (95%CI:1.1–15.8); *p* = 0.04). MVA showed a trend towards worse PFS for NLR-change to <5 (*p* = 0.11). NLR-change to <5 led to non-significant shorter median OS (seven versus 16 months) (HR: 2.3 (95%CI:0.7–7.1); *p* = 0.15). MVA showed non-significant difference for OS. We concluded baseline NLR <5 is associated with improved survival. In contrast, in patients with baseline NLR >5, NLR-change to <5 after eight weeks of Abiraterone was associated with worse survival and should be interpreted carefully.

## 1. Introduction

Lately, several agents were approved for treatment of metastatic castration-resistant prostate cancer (mCRPC). These include, prior to Docetaxel chemotherapy, Abiraterone [1], Enzalutamide [2] and Sipuleucel-T [3], or after Docetaxel chemotherapy, Abiraterone [4], Enzalutamide [5] and Cabazitaxel [6], or when no chemotherapy is indicated in bone-predominant mCRPC without visceral metastases, radium-223 [7]. All of these medications prolong overall survival (OS). However, there is a shortage of available biomarkers to predict response to each therapy and no data concerning the optimal sequence of therapies. Therefore, the decision which medication to choose is largely based on empirical data, experience, or matters like drug availability or the possibility of reimbursement. Further, early imaging, especially in bone mCRPC can be misleading due to the bone flare phenomenon which can represent a response to treatment, although suggesting progressive disease (PD) [8].

Inflammation is recognized to be a relevant driver of cancer progression [9]. The neutrophil-to-lymphocyte ratio (NLR) as an indicator of cancer-related inflammation has been shown to be predictive for treatment response or even suggested as a marker of therapy control in various tumour entities, for example in renal cell cancer, oesophageal cancer, gastrointestinal cancer, and many others [10,11,12,13]. In mCRPC patients treated with Ketoconazole, a NLR cut-off of <3 was identified for differentiation between patients with longer or shorter progression-free survival (PFS) [14]. In the SUN-1120-trial on Sunitinib vs. Placebo in mCRPC Sonpavde found that a NLR > 2.5 and even more so >5 was prognostic of poor OS [15]. And in a post hoc analysis of men treated with Docetaxel within the TAX-327 and VENICE-trials a NLR >than the median of 2, 2.1 was associated with worse survival [16]. A retrospective study on mCRPC patients treated with Abiraterone identified a baseline NLR of <5 in combination with the extent of metastatic spread to be associated with prostate-specific antigen (PSA) response, and overall survival [17].

We hypothesize that in mCRPC patients treated with Abiraterone, the NLR might not only be useable as a predictor of treatment response at baseline, but also as a marker of early treatment response.

## 2. Results

### 2.1. Patient Characteristics

The descriptive characteristics of the cohort are presented in Table 1. The study cohort consisted of 96 mCRPC patients, of whom 52 were treated within the pre- and 44 in the post-Docetaxel setting. The median follow-up was 20.0 months (interquartile range (IQR): 11.0–28.0). The median time of Abiraterone treatment was 10.0 months (IQR: 6.0–14.3). No dose modification was necessary for any patient. The median age of the patients was 70.0 years (IQR: 62.0–76.3). Visceral metastases were present in 28.1% at the beginning of Abiraterone treatment. An unfavourable Gleason-Score of ≥8 was seen in 59.8% of the men. The median baseline values of PSA, alkaline phosphatase (ALP) and LDH (lactate dehydrogenase) were 134 ng/mL (IQR: 45–349), 126 U/L (IQR: 86–296) and 251 U/L (IQR: 210–358), respectively.

At baseline, a NLR of <5 was present in 79 (82.3%) of the patients. Compared with men with a NLR >5, patients with a NLR <5 had a significantly lower median baseline PSA-level (91 vs. 224 ng/mL; Mann-Whitney *U*-test: *p* = 0.04) and lower baseline median LDH-levels (240 vs. 308 U/L; *p* = 0.04). Further, the baseline LDH was elevated above the upper limit of normal (ULN) at baseline in a larger proportion in the men with a NLR >5 (88.2% vs. 59.5%; *χ*^2^-test: *p* = 0.03). The other baseline characteristics were not statistically different. Bone protective medication (Denosumab or Zoledronic acid) was well balanced between both groups (58.8% vs. 59.5%; *χ*^2^-test: *p* = 0.96) (Table 1).

According to prostate cancer working group 2 (PCWG2)-criteria, PSA-response is defined as a PSA decline of ≥50% and was seen in 35.4% of the patients with a baseline NLR >5 compared with 53.2% of the men with a baseline NLR <5 (*χ*^2^-test: *p* = 0.18). A decline of ≥90% was found in 5.9% vs. 27.8% (*χ*^2^-test: *p* = 0.06).

### 2.2. Prognostication of Survival Outcomes at Baseline

Results of univariate and multivariate analyses for PFS and OS based on baseline NLR (>5 vs. <5) are displayed in Table 2, Table 3 and Table 4.

In univariate analysis (Table 2), baseline LDH >ULN (hazard ratio (HR): 2.6 (95% confidence interval (95%CI): 1.6–4.3); *p* < 0.01), Eastern Collaborative Oncology Group performance status (ECOG) ≥ 2 (HR: 2.9 (95%CI: 1.7–4.9); *p* < 0.01), and the presence of visceral metastases (HR: 2.2 (95%CI: 1.4–3.5); *p* = 0.02) were associated with worse PFS.

**Figure 1:**
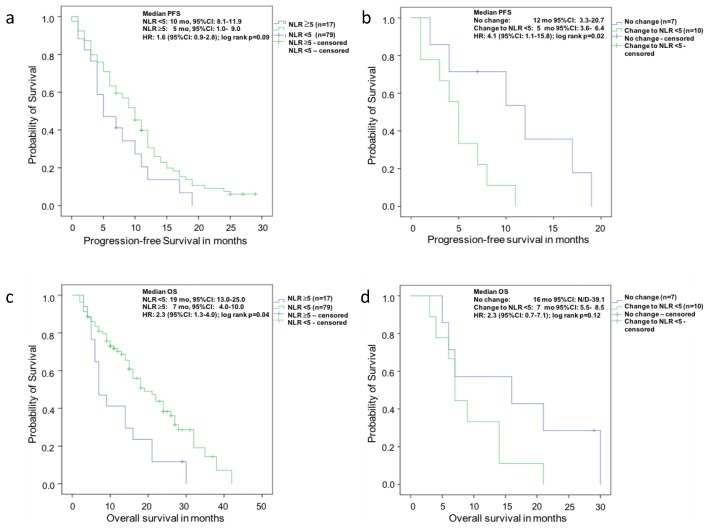
Kaplan-Meier analysis for progression-free survival probability of patients with mCRPC under therapy with Abiraterone (**a**) with baseline neutrophil-to-lymphocyte ratio (NLR) < 5 vs. ≥5; (**b**) Patients with or without change of NLR to <5 after eight weeks of therapy and (**c**) overall survival probability with baseline NLR < 5 vs. ≥5; and (**d**) patients with or without change of NLR to <5 after eight weeks of therapy.

At baseline there was a trend for improved median PFS of patients with a NLR <5 (10 months (95%CI: 8.1–11.9)) compared with men with a NLR >5 (five months (95%CI: 1.0–9.0), log-rank *p* = 0.09) (Figure 1a).

After adjusting for the parameters significant in univariate analysis and clinically-important variables, ECOG ≥2 (HR: 2.6 (95%CI: 1.5–4.5); *p* = 0.01), baseline LDH >UNL (HR: 2.2 (95%CI: 1.3–3.8); *p* = 0.01) and the presence of visceral metastases (HR: 1.7 (95%CI: 1.0–2.9); *p* = 0.04) remained independent prognosticators of poor PFS (Table 4).

The univariate analysis for OS showed that a baseline NLR >5 (HR: 2.3 (95%CI: 1.3–4.0); *p* < 0.01), ECOG ≥2 (HR: 3.4 (95%CI: 1.9–6.0); *p* < 0.01), baseline LDH >UNL (HR: 3.1 (95%CI: 1.7–5.8); *p* < 0.01) and baseline ALP >UNL (HR: 1.9 (95%CI: 1.2–3.2); *p* < 0.01) were associated with worse OS (Table 3).

In Kaplan-Meier analysis the median OS (Figure 1c) of men with a baseline NLR <5 (19 months (95%CI 13.0–25.0) was significantly longer than in patients with a NLR >5 (seven months (95%CI: 4.0–10.0), log-rank *p* = 0.04).

In multivariate analysis of OS (Table 4), only ECOG ≥2 (HR: 3.0 (95%CI: 1.6–5.5); *p* < 0.01) and baseline LDH >UNL (HR: 2.4 (95%CI: 1.2–4.6); *p* = 0.01) remained independent prognosticators of worse survival. For NLR >5, OS was reduced but not significantly (HR: 1.7 (95%CI: 0.9–3.0); *p* = 0.10).

### 2.3. Prognostication of Survival Outcomes with Change of Neutrophil-to-Lymphocyte Ratio (NLR) to <5 in Patients with a Baseline NLR > 5 vs. no Change after Eight Weeks of Abiraterone

Seventeen men (17.7%) of the whole cohort had a NLR >5 at baseline. In the patients with a change of NLR from >5 to <5 after eight weeks of treatment with Abiraterone (*n* = 10) the neutrophils declined in all but one of the cases in which the lymphocytes increased. Seven patients remained with an NLR of >5 after eight weeks of therapy.

For PFS the univariate Cox-regression analysis (Table 2) showed that change of NLR to <5 at eight weeks of Abiraterone therapy was associated with worse survival (HR: 4.1 (95%CI: 1.1–15.8); *p* = 0.04). All other variables, including decline of PSA ≥50%, were no significant prognosticators.

In the Kaplan-Meier analysis of PFS (Figure 1b), equally, the median survival was in favour of no change of NLR (12 months (95%CI 3.3–20.7) compared to change to NLR <5 (five months (95%CI 3.6–6.4), log-rank *p* = 0.02)).

After adjusting for decline of PSA ≥50%, there was only a trend for prognostication of worse PFS for change of NLR to <5 (HR: 3.4 (95%CI: 0.8–15.2); *p* = 0.11) (Table 4).

The univariate analysis of OS (Table 3) showed a trend for change of NLR to <5 (HR: 2.3 (95%CI: 0.7–7.1); *p* = 0.15) and no decline of PSA ≥50% (HR: 2.9 (95%CI: 0.9–9.4); *p* = 0.08) towards poor survival. 

In Kaplan-Meier analysis of OS (Figure 1d) the change of NLR to <5 was associated with a shorter survival of 7 months (95%CI 5.5–8.5) compared with 16 months (95%CI N/D-39.1) in patients with no change of NLR. However, this difference was not statistically significant (log-rank *p* = 0.12).

In multivariate Cox-regression analysis (Table 4) there was no significant difference for the prognostication of worse OS in patients with change of NLR to <5 and no decline of PSA ≥50% (*p* = 0.47 and 0.25, respectively).

**Figure 2:**
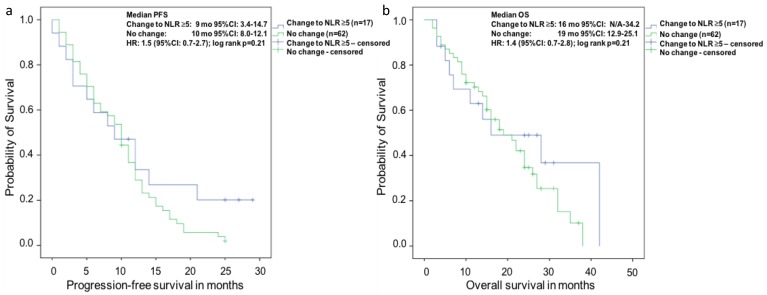
Kaplan-Meier analysis of the subgroup of patients with a NLR <5 at baseline for (**a**) overall survival (OS) and (**b**) progression-free survival (PFS). There was no difference of median survival in the subgroup of patients with a NLR <5 at baseline and change of NLR to >5 vs. no change after eight weeks of Abiraterone therapy (log rank *p* = 0.21). There equally was no difference of median PFS for change of NLR to >5 vs. no change after eight weeks of Abiraterone therapy (log rank *p* = 0.21).

In Kaplan-Meier analysis of the 79 patients with an initial NLR of <5 at baseline (Figure 2) there was no difference between the subgroup of patients with change of NLR to >5 and in the subgroup with no change of NLR for both PFS and OS (log rank *p* = 0.21, respectively).

## 3. Discussion

The NLR was found to be prognostic for survival outcomes and recurrence rate in various tumour entities [18,19]. For prostate cancer, especially in mCRPC, the NLR was equally shown to be prognostic for survival [10,17]. In a study on mCRPC patients treated with Abiraterone, Leibowitz-Amit et al. found that a composite score of baseline NLR <5 and extent of metastatic spread was associated with PSA-response [17]. Our results support these findings. In our cohort of mCRPC patients treated with Abiraterone the baseline NLR with a cut-off of <5 without addition of metastatic spread equally was a prognosticator for prolonged OS. However, after adjustment for other important variables in multivariate analysis there was only a trend towards statistical significance regarding survival. Maybe the limited number of patients with a baseline NLR >5 (*n* = 17) included in this study, precluded reaching significant values in multivariate analyses for survival endpoints.

The underlying immunologic mechanisms are still unclear, but most likely the interactions between tumour cells and immunologic cells and mediators undergo specific changes [9]. An elevated (expected unfavourable) NLR can be caused by rising neutrophils or declining lymphocytes or both. Assuming that a high baseline NLR is prognostic for outcomes under given treatments it would be reasonable to presume that a change of an elevated (expected unfavourable) NLR to a lower (potentially favourable) NLR under treatment could be useful to identify patients with exceptionally good and lasting responses to therapy.

So far, there is very little evidence on NLR as a therapy control marker. For mCRPC there is only one study by Lorente et al., which addressed this matter [10]. Here, in a post hoc analysis of the large registration trial of Cabazitaxel vs. Mitoxantrone, a baseline NLR of <3 was identified to be associated with better survival, better PSA-response and better objective response rate according to RECIST-criteria. Our study is the first to address change of NLR as a treatment response marker in patients treated with Abiraterone. Interestingly, in that study the conversion of an unfavourable NLR of >3 to a favourable NLR < 3 under therapy was independently associated with an improved OS. This result was in line with the expectable. In contrast and most surprisingly, in our study we found that a change of a supposedly unfavourable NLR > 5 to the expected to be favourable NLR <5 after eight weeks of Abiraterone therapy in mCRPC patients was associated with both worse PFS and OS. The reasons for these findings are unclear. One possible explanation could be the immunomodulating effect of Prednisone, which has to be administered to prevent mineral corticoid excess under Abiraterone therapy. In our study, all patients received the recommended dose of 5 mg Prednisone bi-daily with the start of Abiraterone, but no patient had prior prednisone therapy. In the trial of Lorente et al. [10] 45% of the patients had already been on a stable dose of a corticosteroid before initiation of Cabazitaxel-/Mitoxantron-therapy and, thus, at the time when the baseline NLR was determined. The subgroup analysis of patients with or without baseline Prednisone use showed that the patients with Prednisone use had a significantly higher NLR of 3.9 vs. 2.9 (*p* < 0.001) and patients with NLR > 3 were more likely to receive baseline Prednisone (*p* = 0.016). However, Lorente et al. tested for confounding variables and showed that the NLR < 3 was predictive of survival with or without corticosteroid use. This basically supports the role of corticosteroids for elevating the NLR in general. In our study all patients had been treated with Prednisone starting with initiation of Abiraterone therapy. This difference could explain our unexpected findings. Prednisone physiologically leads to an increase of neutrophils first by about 20% by demarginalisation of the intravascular pool, and consecutively, by a greater increase caused by mobilization of the bone marrow neutrophil reserve [20]. Naturally, this effect would be evident for all patients treated with Prednisone and Abiraterone. In the subset of patients with a change of NLR from >5 to <5 after eight weeks of Abiraterone therapy in our study the neutrophils declined in almost all of the cases. An explanation for this may be that these patients had a higher tumour load especially of bone metastases thus leading to a depletion of functioning bone marrow with the production of neutrophils most likely being impaired by bone marrow insufficiency as a result of tumour progression under Abiraterone. The immunomodulating effect of Prednisone under Abiraterone may have a stronger impact due to increasingly depleted bone marrow reserves in case of disease progression.

Recently there were findings that PSA change within four weeks after the start of therapy may be prognostic for survival outcomes. For example, Facchini et al. showed that PSA-change after 15 days of Abiraterone was a surrogate for PFS and OS [21]. Therefore, for future studies, research on change of NLR and the possibility of complement of PSA and change of NLR in this very early setting may be promising.

Our study is limited by problems inherent to the retrospective approach and a relatively small sample size. This holds especially true for the subset of patients with change of NLR to <5. Therefore, our findings need to be validated by prospective studies. The single parameter NLR cut-off of less than, or greater than five needs to be validated as well. We did not do imaging in a per protocol routine fashion and, therefore, could not calculate radiographic PFS.

The underlying effects of the NLR and changes of NLR in relationship to cancer are still unclear and especially our findings concerning unexpected changes under therapy with Prednisone/Abiraterone need to be elucidated.

## 4. Materials and Methods

### 4.1. Patients

After ethics committee-approval (2007-467-f-S), we retrospectively reviewed all patients with mCRPC presenting at the Department of Urology in the Muenster University Medical Center between December 2009 and July 2015 and receiving Abiraterone to analyse the impact of the NLR on progression-free and overall survival. All patients gave written informed consent before participating. We performed the study according to the declaration of Helsinki.

The study cohort consisted of confirmed mCRPC patients as defined by PCWG2-criteria [22] in pre- or post-chemotherapy setting. All patients met the requirements for Abiraterone-treatment. The men receiving Abiraterone prior to Docetaxel all had to be asymptomatic or oligo-symptomatic with no need to use opiates and degree of pain of no more than three out of 10 on the numeric-rating-scale. Patients receiving Abiraterone after Docetaxel all had progressive disease on or after chemotherapy. During the reviewed timeframe a total of 96 patients presented with evaluable NLR-data of which 52 patients were treated prior to, and 44 after, chemotherapy, respectively. Five patients (4.8%) had received Enzalutamide prior to Abiraterone. All patients included in the analysis were on a stable dose of anti-bone resorptive medication (Denosumab or Zoledronic acid) at least three months prior to the start of Abiraterone, and during the whole treatment phase, or did not receive antiresorptive medication at all.

The day before start of Abiraterone therapy all patients had blood drawn for baseline analysis of PSA, neutrophils, lymphocytes, and serum chemistry. The analysis of PSA was repeated two and four weeks after initiation of therapy, and four weekly thereafter. Neutrophils and lymphocyte measurements were repeated after eight weeks. PSA-progression was defined according to PCWG2-criteria, i.e., PSA-progression at the date that a 25% or greater increase and an absolute increase of 2 ng/mL or more from the nadir is documented, which is confirmed by a second value obtained three or more weeks later [22].

For documentation of baseline values and changes of soft tissue metastases CT- or MRI-scans of thorax, abdomen and pelvis were performed. For information on bone metastases bone scans were used. Imaging was repeated during the course of the Abiraterone-therapy whenever clinically indicated but not in routine fashion. PD was defined according to RECIST 1.1 criteria for axial cross sectional imaging [23] and by PCWG2 criteria for bone scans [22].

The current response status, i.e., complete remission (CR), partial remission (PR), stable disease (SD) or PD was assessed after each visit. PD was considered evident upon deterioration of general condition or worsening of pain when unequivocally caused be prostate cancer, when PSA-progression occurred or when radiographic progression was detected. The baseline cohort was subdivided according to NLR (group-1 (favorable): NLR < 5; group-2 (non favorable): NLR > 5).

### 4.2. Statistical Methods

For descriptive statistics we report medians with 95% CI or IQR for continuous variables and as populations and frequencies for categorical variables. We used the *χ*^2^-test, Fisher’s exact-test or Mann-Whitney *U*-test for determining the significance of differences between categorical and continuous variables. For analysis of survival outcomes were applied Kaplan-Meier-analysis. For uni- and multivariate analysis of significance for survival outcomes we used Cox-regression-models. All reported *p*-values are two-sided and we assumed statistical significance when *p* ≤0.05. We used SPSS-Statistics V.23 (IBM Inc., Armonk, NY, USA) for statistical assessment.

## 5. Conclusions

In patients with mCRPC under Abiraterone treatment a pre-treatment NLR < 5 is associated with better survival outcomes. Unexpectedly, after eight weeks of Abiraterone therapy, a change of initially elevated NLR of >5 to <5 was associated with worse survival. The immunomodulating effect of concomitant Prednisone next to Abiraterone combined with insufficient bone marrow reserves in patients with advanced (bony) disease may be the reason for this phenomenon. Thus, a putatively favourable change of NLR to <5 may represent worsening of disease and should be interpreted cautiously. A deeper understanding of the underlying immune mechanisms in this setting is highly warranted. Therefore, larger prospective trials on the matter are needed prior to application of change of NLR in mCRPC.

## Figures and Tables

**Table 1:** Characteristics of patients with metastatic castration-resistant prostate cancer (mCRPC) on Abiraterone with a neutrophil-to-lymphocyte-ratio (NLR) <5 and >5.

Variable	All	NLR <5	NLR >5	*p*
Patients (*n*) (%)	96	79 (82.3)	17 (17.7)	
Median NLR Baseline (ng/mL) (IQR)	3.2 (2.5–4.5)			
Age, median (years) (IQR)	70.0 (63.0–76.3)	70.0 (62.0–76.0)	70.0 (65.0–78.0)	0.60
Median follow-up	20.0 (11.0–28.0)	17.5 (11.0–26.8)	29.0 (29.0–29.0)	0.28
Median duration AA therapy (months) (IQR)	10.0 (6.0–14.3)	11.0 (7.0–15.0)	7.0 (5.0–11.5)	0.08
GS ≥8 (*n*) (%)	55 (59.8)	46 (60.5)	9 (56.3)	0.75
Lnn. Metastases (*n*) (%)	61 (63.5)	50 (63.3)	11 (64.7)	0.91
Visceral Metastases (*n*) (%)	27 (28.1)	24 (30.4)	3 (17.6)	0.38
Bone Metastases (*n*) (%)	85 (88.5)	69 (87.3)	16 (94.1)	0.68
Pre CTX (*n*) (%)	52 (54.2)	44 (55.7)	8 (47.1)	0.52
Post CTX (*n*) (%)	44 (45.8)	35 (44.3)	9 (52.9)	
Patients died (*n*) (%)	67 (69.8)	51 (64.6)	16 (94.1)	0.02
ECOG (all) (*n*) (%)				
0	19 (20.0)	16 (20.5)	3 (17.6)	0.91
1	57 (60.0)	47 (60.3)	10 (58.8)	
2	19 (20.0)	15 (19.2)	4 (23.5)	
Antiresorptive therapy (*n*) (%)	57 (59.4)	47 (59.5)	10 (58.8)	0.96
Zoledronic acid (*n*) (%)	40 (41.7)	33 (41.8)	7 (41.2)	0.96
Denosumab (*n*) (%)	19 (19.8)	15 (19.0)	4 (23.5)	0.67
Best clinical outcome (*n*) (%)				
CR	1 (1.1)	1 (1.3)	0 (0)	0.61
PR	54 (56.8)	46 (59.0)	8 (47.1)	
SD	27 (28.4)	20 (25.6)	7 (41.2)	
PD	13 (13.7)	11 (14.1)	2 (11.8)	
PSA red. ≥50% (*n*) (%)	48 (50.0)	42 (53.2)	6 (35.3)	0.18
PSA red. ≥90% (*n*) (%)	23 (24.0)	22 (27.8)	1 (5.9)	0.06
Median PSA Baseline (ng/mL) (IQR)	134 (45–349)	91 (35–334)	224 (107–589)	0.04
Median LDH Baseline (U/L) (IQR)	251 (210–358)	240 (205–357)	308 (245–386)	0.04
LDH BL >UNL (*n*) (%)	62 (64.6)	47 (59.5)	15 (88.2)	0.03
Median ALP Baseline (U/L) (IQR)	126 (86–296)	126 (91–297)	103 (77–266)	0.43

Abbreviations: NLR: neutrophil-to-lymphocyte ratio; IQR: interquartile range; Lnn: lymphonodal; CTX: chemotherapy; CR: complete remission; PR: partial remission; SD: stable disease; PD: progressive disease; ECOG: Easter collaborative Oncology Group; GS: Gleason score; PSA: prostate specific antigen; LDH: lactate dehydrogenase; ALP: alkaline phosphatase; BL: baseline; UNL: upper normal limit.

**Table 2:** Univariate analysis for baseline biomarkers for progression-free survival in (a) 96 mCRPC-patients prior to Abiraterone therapy and (b) in 17 patients with a baseline NLR >5 after eight weeks of therapy.

(a) Univariate Analysis PFS at Baseline			(b) Univariate Analysis of PFS in *n* = 17 Patients with Baseline NLR > 5 after Eight Weeks of Abiraterone		
Variable	HR (95%CI)	*p*	Variable	HR (95%CI)	*p*
ECOG		<0.01	ECOG		0.57
0–1	1 (reference)		0–1	1 (reference)	
2	2.9 (1.7–4.9)		2	1.4 (0.4–4.8)	
LDH baseline >UNL		<0.01	LDH baseline >UNL	1 (reference)	0.52
No	1 (reference)		No		
Yes	2.6 (1.6–4.3)		Yes	2.0 (0.3–15.4)	
Visceral metastases		0.02	Visceral metastases		0.85
No	1 (reference)		No	1 (reference)	
Yes	2.2 (1.4–3.5)		Yes	0.9 (0.2–3.3)	
ALP baseline >UNL		0.13	ALP baseline >UNL		0.55
No	1 (reference)		No	1 (reference)	
Yes	1.4 (0.9–2.1)		Yes	1.4 (0.5–3.9)	
NLR		0.11	NLR		0.04
<5	1 (reference)		No change	1 (reference)	
>5	1.6 (0.9–2.8)		Change to <5	4.1 (1.1–15.8)	
Abiraterone		0.10	PSA decline ≥50%		0.14
Pre-Docetaxel	1 (reference)		Yes	1 (reference)	
Post-Docetaxel	1.4 (0.9–2.2)		No	2.7 (0.7–10.2)	
Gleason Score		0.10	Abiraterone		0.41
<8	1 (reference)		Pre-Docetaxel	1 (reference)	
≥8	1.5 (0.9–2.3)		Post-Docetaxel	1.6 (0.5–4.6)	
Lymphonodal metastases		0.77	Gleason Score		0.63
No	1 (reference)		<8	1 (reference)	
Yes	0.9 (0.6–1.5)		≥8	1.3 (0.4–3.9)	
Bone Metastases		0.82	Lymphonodal metastases		0.41
No	1 (reference)		No	1 (reference)	
Yes	1.1 (0.5–2.2)		Yes	0.6 (0.2–1.9)	

Abbreviations: PFS: progression free survival; HR: hazard ratio; 95%CI: 95% confidence interval; ECOG: Eastern Collaborative Oncology Group; LDH: lactate dehydrogenase; UNL: upper normal limit; PSA: prostate-specific antigen; NLR: neutrophil-to-lymphocyte ratio; ALP: alkaline phosphatase; OS: overall survival.

**Table 3:** Univariate analysis for baseline biomarkers for overall survival in (a) 96 mCRPC-patients prior to Abiraterone therapy and (b) in 17 patients with a baseline NLR >5 after eight weeks of therapy.

(a) Univariate Analysis OS at Baseline			(b) Univariate Analysis of OS in *n* = 17 Patients with Baseline NLR >5 after Eight Weeks of Abiraterone		
Variable	HR (95%CI)	*p*	Variable	HR (95%CI)	*p*
ECOG		<0.01	ECOG		0.60
0–1	1 (reference)		0–1	1 (reference)	
2	3.4 (1.9–6.0)		2	1.4 (0.3–4.5)	
LDH baseline >UNL		<0.01	LDH baseline >UNL		0.65
No	1 (reference)		No	1 (reference)	
Yes	3.1 (1.7–5.8)		Yes	0.7 (0.2–3.3)	
Visceral metastases		0.35	Visceral metastases		0.76
No	1 (reference)		No	1 (reference)	
Yes	1.3 (0.8–2.2)		Yes	0.8 (0.2–2.9)	
ALP baseline >UNL		<0.01	ALP baseline >UNL		0.83
No	1 (reference)		No	1 (reference)	
Yes	1.9 (1.2–3.2)		Yes	0.9 (0.3–2.5)	
NLR		<0.01	NLR		0.15
<5	1 (reference)		No change	1 (reference)	
>5	2.3 (1.3–4.0)		Change to <5	2.3 (0.7–7.1)	
Abiraterone		0.06	PSA decline ≥50%		0.08
Pre-Docetaxel	1 (reference)		Yes	1 (reference)	
Post-Docetaxel	1.6 (1.0–2.6)		No	2.9 (0.9–9.4)	
Gleason Score		0.67	Abiraterone		0.38
<8	1 (reference)		Pre-Docetaxel	1 (reference)	
≥8	1.1 (0.7–1.9)		Post-Docetaxel	1.6 (0.6–4.6)	
Lymphonodal metastases		0.35	Gleason Score		0.93
No	1 (reference)		<8	1 (reference)	
Yes	0.8 (0.5–1.3)		≥8	1.1 (0.4–3.0)	
Bone metastases		0.62	Lymphonodal metastases		0.64
No	1 (reference)		No	1 (reference)	
Yes	1.3 (0.5–3.2)		Yes	0.8 (0.3–2.2)	

Abbreviations: PFS: progression-free survival; HR: hazard ratio; 95%CI: 95% confidence interval; ECOG: Eastern Collaborative Oncology Group; LDH: lactate dehydrogenase; UNL: upper normal limit; PSA: prostate-specific antigen; NLR: neutrophil-to-lymphocyte ratio; ALP: alkaline phosphatase; OS: overall survival.

**Table 4:** Multivariate analysis for significant baseline biomarkers for (a) progression-free and (b) overall survival in 96 mCRPC-patients prior to Abiraterone therapy and (c) progression-free survival and (d) overall survival in 17 patients with a baseline NLR >5 after eight weeks of therapy.

**(a) Multivariate Analysis PFS at Baseline**			**(c) Multivariate analysis of PFS in *n* =17 Patients with Baseline NLR >5 after Eight Weeks of Abiraterone**		
**Variable**	**HR (95%CI)**	***p***	**Variable**	**HR (95%CI)**	***p***
ECOG		0.01	NLR		0.11
0–1	1 (reference)		No change	1 (reference)	
2	2.6 (1.5–4.5)		Change to <5	3.4 (0.8–15.2)	
LDH baseline >UNL		0.01	PSA decline ≥50%		0.14
No	1 (reference)		Yes	1 (reference)	
Yes	2.2 (1.3–3.8)		No	1.5 (0.3–6.4)	
Visceral metastases		0.04			
No	1 (reference)				
Yes	1.7 (1.0–2.9)				
ALP baseline >UNL		0.25			
No	1 (reference)				
Yes	1.3 (0.8–2.1)				
NLR		0.71			
<5	1 (reference)				
>5	1.1 (0.6–2.0)				
**(b) Multivariate analysis OS at baseline**			**(d) Multivariate Analysis of OS in *n* = 17 Patients with Baseline NLR >5 after Eight Weeks of Abiraterone**		
**Variable**	**HR (95%CI)**	***p***	**Variable**	**HR (95%CI)**	***p***
ECOG		<0.01	NLR		0.47
0–1	1 (reference)		No change	1 (reference)	
2	3.0 (1.6–5.5)		Change to <5	1.6 (0.5–5.6)	
LDH baseline >UNL		0.01	PSA decline ≥50%		0.25
No	1 (reference)		Yes	1 (reference)	
Yes	2.4 (1.2–4.6)		No	2.2 (0.6–8.1)	
Visceral metastases		0.56			
No	1 (reference)				
Yes	1.2 (0.7–2.1)				
ALP baseline >UNL		0.05			
No	1 (reference)				
Yes	1.7 (1.0–2.9)				
NLR		0.10			
<5	1 (reference)				
>5	1.7 (0.9–3.0)				

Abbreviations: PFS: progression free survival; HR: hazard ratio; 95%CI: 95% confidence interval; ECOG: Eastern Collaborative Oncology Group; LDH: lactate dehydrogenase; UNL: upper normal limit; PSA: prostate-specific antigen; NLR: neutrophil-to-lymphocyte ratio; ALP: alkaline phosphatase; OS: overall survival.

## Abbreviations

mCRPC	Metastatic castration resistant prostate cancer
OS	Overall survival
PD	Progressive disease
NLR	Neutrophil-to-lymphocyte ratio
PFS	Progression-free survival
PSA	Prostate specific antigen
IQR	Interquartile range
ALP	Alkaline phosphatase
LDH	Lactate dehydrogenase
ULN	Upper normal limit
PCWG2	Prostate cancer working group 2
HR	Hazard ratio
95%CI	95% confidence interval
ECOG	Eastern Collaborative Oncology Group Performance Status
CR	Complete remission
PR	Partial remission
SD	Stable disease
